# Clinical immunogenicity assessment of HIV/AIDS vaccines: current progress and future perspectives

**DOI:** 10.1080/22221751.2026.2751675

**Published:** 2026-10-17

**Authors:** Hang Yu, Xi Wu, Tingting Li, Jianhui Nie

**Affiliations:** a Division of HIV/AIDS and Sex-Transmitted Virus Vaccines, National Institutes for Food and Drug Control (NIFDC), State Key Laboratory of Drug Regulatory Science, NHC Key Laboratory of Research on Quality and Standardization of Biotech Products, NMPA Key Laboratory for Quality Research and Evaluation of Biological Products, Beijing, People’s Republic of China; b School of Pharmacy, China Pharmaceutical University, Nanjing, People’s Republic of China

**Keywords:** HIV vaccine, immunogenicity, evaluation methods, correlates of protection, clinical trials

## Abstract

Developing a safe and effective HIV-1 vaccine remains the cornerstone of global efforts to terminate the AIDS pandemic. However, the virus's immense genetic diversity and the persistent absence of a universal Correlate of Protection (CoP) present formidable barriers. Concomitant with the iterative evolution of vaccine design – spanning from protein subunits to mRNA and germline-targeting platforms – immunogenicity evaluation frameworks have undergone a critical paradigm shift. The field has transitioned from relying on conventional serological assays to embracing comprehensive, high-dimensional immune profiling. This review delineates the trajectory of milestone clinical trials, including RV144, the HVTN series, and the AMP studies, to systematically summarize advancements in assessing both humoral and cellular immunity. We comprehensively examine core methodologies, such as Binding Antibody Multiplex Assays (BAMA), pseudovirus neutralization, Enzyme-Linked Immunospot (ELISpot), and Intracellular Cytokine Staining (ICS). Furthermore, we highlight a pivotal transition in evaluative logic: moving beyond a singular focus on neutralizing antibody titres to integrate Fc-mediated effector functions (e.g. Antibody-Dependent Cellular Cytotoxicity (ADCC) and Phagocytosis (ADCP)) and the synergistic roles of polyfunctional T-cells. We also explore the transformative potential of burgeoning frontiers, including systems serology, single-cell B-cell receptor (BCR) sequencing, and mucosal immunity evaluation. Ultimately, we recommend that future evaluation frameworks combine single-cell multi-omics technologies with artificial intelligence algorithms and integrate multi-dimensional data for analysis, thereby providing a solid theoretical and technical foundation for the design of next-generation HIV vaccines.

## Introduction

### An overview of HIV/AIDS

Acquired Immunodeficiency Syndrome (AIDS), caused by the Human Immunodeficiency Virus (HIV), manifests as profound immunodeficiency, opportunistic infections, and malignancies. Epidemiologically, HIV-1 remains a lethal pandemic transmitted via sexual, blood, or vertical routes, with transmission efficiency tied to viral load. Its prolonged latency facilitates asymptomatic transmission. Despite its zoonotic origin, urbanization and migration have driven its global spread [1].

HIV, a *Retroviridae* lentivirus, comprises HIV-1 and HIV-2. HIV-1 pathogenesis involves persistent replication, selectively depleting CD4^+^ T lymphocytes [2], causing systemic dysfunction across the hematopoietic and nervous systems [3]. Without antiretroviral therapy (ART), HIV-1 maintains a high-turnover equilibrium, releasing 10^10^ virions daily [4]. This prolific replication, coupled with error-prone reverse transcription, drives extreme genomic mutability, posing a formidable biological challenge [5]. Furthermore, HIV-1 integrates into host cell chromosomes to establish stable, transcriptionally silent reservoirs. While resting memory CD4^+^ T cells remain a primary and classical component, current research recognizes that viral reservoirs also encompass specialized T cell subsets such as T follicular helper (T_fh_) cells and stem-cell-like memory T (T_SCM_) cells, tissue-resident macrophages, and anatomical compartments including lymph nodes and gut-associated lymphoid tissue [6]. Given the 44-month half-life of these cells, ART-mediated eradication is estimated to require more than 70 years, rendering a cure impossible without total proviral DNA clearance [7]. Because these complex biological characteristics and stable viral reservoirs make complete eradication unachievable with current therapies, the development of a safe, effective HIV vaccine remains the ultimate strategy for global prevention and control.

### Historical and current landscape of HIV vaccine development

Initial strategies targeting either humoral or cellular immunity failed: gp120 subunit trials (VAX004/VAX003) lacked neutralizing efficacy against dominant strains such as CRF01_AE [8], and rAd5-vectored T-cell vaccines (STEP, HVTN 505) failed to prevent infection or reduce viral loads [9,10]. Notably, pre-existing Ad5 immunity in STEP inadvertently increased HIV acquisition in uncircumcised men by stimulating the dendritic-T cell axis [11,12], underscoring the critical importance of vector selection and immune activation [13].

The RV144 trial provided the first human proof-of-concept with a 31.2% protection rate [14]. Protection correlated with V1V2-specific IgG, while elevated plasma envelope (Env)-specific IgA counteracted these benefits [15], highlighting the importance of non-neutralizing effector functions over conventional neutralizing breadth. To adapt this CRF01_AE-specific regimen for subtype C in Africa, the “P5” partnership launched HVTN 100 and HVTN 702 [16]. Despite optimizations (subtype C immunogens, MF59 adjuvant, additional boosters) yielding favourable Phase 1/2 immunogenicity [17,18], the Phase 2b/3 HVTN 702 trial failed due to inadequate, transient V1V2-specific IgG, proving that simply altering clades or adjuvants cannot easily translate protection across populations [19,20].

To counter HIV-1 hypermutability, Ad26-vectored “mosaic” vaccines were engineered. Despite non-human primate success [21], the Imbokodo [22,23] and Mosaico [24] trials failed to protect high-risk cohorts. Conversely, the HVTN 124 DNA-prime/protein-boost regimen elicited superior V1V2 IgG3 and CD4^+^ T-cell responses, positioning it as a leading candidate [25,26]. Meanwhile, the Antibody Mediated Prevention (AMP) trials (HVTN 704/703) demonstrated that passive immunization with the broadly neutralizing antibody (bnAb) VRC01 provided 75.4% efficacy against sensitive strains. However, because VRC01 covered only approximately 30% of circulating global strains, VRC01 monotherapy failed to significantly reduce overall HIV-1 acquisition [27,28]. Extensive computational modelling and preclinical studies have since established that combining multiple non-competing bnAb targeting distinct envelope epitopes is essential to maximize strain coverage, increase potency, and prevent neutralization escape [29].

Building on IAVI G001’s eOD-GT8 nanoparticles priming of CD4-binding site bnAb precursors [30,31], IAVI G002 and G003 expanded germline targeting to mRNA platforms. G002 proved that heterologous mRNA prime-boosting drives somatic hypermutation(SHM) and structural maturation of VRC01-class B cells, while G003 confirmed robust priming efficacy and cross-population consistency in African cohorts [32]. Coupled with HVTN 302’s mRNA-LNP induction of Tier 2 neutralizing antibodies [33,34]. These trials provide landmark clinical proof-of-concept for multi-stage HIV immunization.

Researchers at the Chinese Center for Disease Control and Prevention selected the Tiantan strain of the Chinese smallpox vaccine as a vector for an HIV vaccine and, after attenuating it, developed a replicating Tiantan vaccinia (rTV) HIV vaccine. An immunogenicity study was conducted using a heterologous sequential immunization strategy consisting of an initial DNA vaccine dose followed by a booster dose of the rTV vaccine. Experimental results confirmed that this vaccine combination induces robust humoral and cellular immune responses and protects rhesus monkeys against SHIV infection. Phase I and Phase II clinical trials have been completed [35,36]. The pRhPD1-p27 utilizes the highly conserved SIV/HIV capsid protein Gag-p27 as its immunogen, expressed as a fusion protein with soluble programmed death-1 (PD-1). PD-1 specifically binds to ligands on the surface of dendritic cells (DCs), enabling targeted antigen presentation directly to DCs. In non-human primate animal experiments, this vaccine achieved sustained viral suppression for over 6 years in the absence of combination antiretroviral therapy (cART), demonstrating that enhancing T-cell immunity can achieve a “functional cure” (cART-free control) for HIV [37–39]. Furthermore, persistent viral vector platforms – most notably attenuated human cytomegalovirus (HCMV) – represent a groundbreaking mucosal vaccination strategy aimed at establishing a durable, highly active barrier of tissue-resident effector memory CD8^+^ T cells and tissue-resident memory (T_RM_) within mucosal and lymphatic portals [40]. The leading candidate, VIR-1388, has recently completed Phase I evaluation [41]. Despite translational hurdles regarding pre-existing vector immunity and unconventional Major Histocompatibility Complex (MHC)-restricted presentation, persistent and mucosal-targeting strategies provide an essential cellular counterpart to humoral protection [42]. Ultimately, overcoming genetic diversity, glycan shielding, and insufficient mucosal immunity necessitates the integration of bnAb, non-neutralizing effector functions, and T_RM_ into a multi-tiered defense strategy.

## Taxonomy of clinical immunogenicity evaluation methods

The characterization of immune responses centres on humoral, cellular, and systemic profiles. Table 1 provides a systematic summary of key HIV clinical trials and these immunogenicity evaluation methods. As the foundational step in immune activation, innate defenses are driven by transient inflammatory cytokine bursts from plasmacytoid dendritic cells (pDCs) [43]. These responses contain early viral replication by recruiting phagocytes and cytotoxic effectors during peak viremia [44]. Researchers utilize ELISpot, flow cytometry, and cytokine profiling to quantify these responses, ensuring vaccines activate sensing pathways [45].

**Table 1.** Immunogenicity assessment in pivotal clinical trials of HIV vaccines.

Trial	Years	Core strategy	Key antigen	Population and phase	Immunisation schedule	Immunogenicity evaluation methods	Primary immunogenicity results	Key findings and implications
VAX004	1998–2003	Humoral immune	Bivalent gp120 (subtype B/B) protein subunit	MSM, High-risk Women; Phase 3	0,1,6,12, 18,24,36 month	ELISA, Neutralization Assay	High-titre binding antibodies, Tier-1 neutralization	VAX004: 6% VE (95% CI −17–24); VAX003: 0% VE (95% CI −7–6). Monomeric gp120 induces non-neutralizing Abs ineffective against circulating strains [8,13].
VAX003	1998–2003	Humoral	Bivalent gp120 (subtype B/E) protein subunit	Injection drug users; Phase 3				
STEP (HVTN 502)	2004–2007	Cellular	MRKAd5 HIV-1 gag/pol/nef	MSM; Phase2b	0, 1, 6 month	ELISpot, ICS	75% of vaccinees elicited T-cell responses; Unexpectedly increased mucosal HIV target cells	VE −8%; HR: 1.5; T-cell immunity insufficient to prevent infection; highlights Ad5 vector risk [10,12].
Phambill (HVTN 503)	2007	Cellular	MRKAd5 HIV-1 gag/pol/nef	MSM; Phase 2b	0, 1, 6 month	ELISpot	89% cross-subtype T-cell response (C subtype); No reduction in viral load	At interim analysis: VE –69%; vaccination halted due to STEP trial results [11].
RV144	2003–2009	Prime-Boost	Prime: ALVAC-HIV (vCP1521, B/E); Boost AIDSVAX B/E ()	General community; Phase 3	Prime: 0,1, 3,6 month; Boost: 3,6 month	BAMA, ELISA, ADCC	Protective CoP: IgG V1V2; Potential interference: High IgA	VE 31.2% (95% CI 1.1–52.1), ELISpot not predictive of protection; cautions against T-cell overactivation [14,36].
HVTN 505	2009–2013	DNA/Ad5	Prime: DNA; Boost: DNA + rAd5	MSM, Trans women; Phase 2b	Prime: 0,4,8 week; Boost: 24 week	ICS, ELISA, BAMA, Neutralization Assay	Robust cellular & humoral immunity; Polyfunctional T cells; Negligible Tier-2 neutralization	VE −25%; HR 1.25.Highlights DNA/Ad5 platform limitations and insufficiency of T-cell responses alone for protection [9].
HVTN 100	2015	Prime-Boost	Prime: ALVAC-HIV; Boost: ALVAC-HIV + Bivalent Subtype C gp120/MF59	General; Phase 1b	Primary: Prime:0,1 month; Boost:3,6,12 month Booster: 30 month	BAMA, ICS, Neutralization Assay	Antigen-specific CD4^+^ T cells; Neutralization Tier-2 viruses; Post-booster: Restored IgG binding Abs	Potent humoral immunity induced; Immunological memory established [17,18].
HVTN 702 (Uhambo)	2016–2020	Prime-Boost	Prime: ALVAC-HIV Subtype C; Boost: Bivalent Subtype C gp120/MF59	Adults at risk; Phase 2b/3	Prime: 0, 1 month Boost: 3,6,12 month	BAMA, ADCC, ICS	High anti-subtype C, gp120 binding Abs, Env-specific CD4^+^ T cells; No association with reduced infection risk for: V1V2 Abs, Env IgA, Tier-1 nAb, ADCC, CD4^+^ T-cell responses	HR 1.02 (95% CI 0.81–1.30), VE 0%. High binding antibody levels, no protection [19,20].
Imbokodo (HVTN 705)	2017–2021	Mosaic/Ad26	Prime: Ad26.Mos4.HIV Boost: Ad26 + Clade C gp140	High-risk women; Phase 2b	Prime: 0,3 month Boost: 6,12 month	ADCP, BAMA, PTE analyze	High vaccine-strain phagocytic activity and PTE-specific T-cell responses; IgG3 V1V2 binding breadth inversely correlated with risk; Lack of bnAbs and insufficient phagocytic activity.	At Month7-24: 14% VE (95% CI −22–40); highlights the challenge of inducing protective immunity against diverse HIV variants [22,23].
Mosaico (HVTN 706)	2019–2023	Mosaic/Ad26	Prime: Ad26.Mos4.HIV Boost: Ad26 + Bivalent gp140	MSM, Transgender; Phase 3	Prime: 0,3 month Boost: Ad26 + Ad26 + Bivalent gp140 6,12 month	ELISA, Neutralization Assay; Serological Evaluation	At Mounth7: >99% binding antibody response rate.	VE 0%. Limitations of mosaic immunogens: non-neutralizing antibodies alone are insufficient against high exposure risk and viral diversity [24,38].
AMP Studies (HVTN 703/704)	2016–2021	Passive	VRC01 bnAb	General; Phase 2b	Every 8 weeks intravenously, 10 doses	IC80, PK, PT80	PT80 threshold: >200. Key correlates: PK and nAb titre vs. protection.	Overall ineffective, but 75.4% VE against VRC01-sensitive viruses [27,28].

Note: VE: Vaccine efficacy; HR: Hazard Ratio; CI: confidence interval; MSM: Men who have sex with men; bnAb: broadly neutralizing antibody; Prime-boost: initial vaccination followed by later booster shots to enhance immune response; ALVAC: canarypox virus vector; Ad26/Ad5: adenovirus serotype 26/5 vectors; PTE (Potential T-cell Epitope); IC80: inhibitory concentration; PK: pharmacokinetic; PT80: predicted serum neutralization 80% inhibitory dilution titre.

Following innate priming, humoral immunity evaluation assesses both quantitative and functional antibody profiles. Enzyme-Linked Immunosorbent Assay (ELISA) and Binding Antibody Multiplex Assay (BAMA) monitor antibody dynamics and seroconversion to guide booster strategies [46]. To assess antiviral neutralization, protocols utilize TZM-bl assays against Tier 1 viruses and Tier 2 viruses [47]. Furthermore, following the RV144 trial, Fc-mediated functions, such as Antibody-Dependent Cellular Cytotoxicity (ADCC) and Phagocytosis (ADCP), shifted from exploratory to core endpoints to evaluate non-neutralizing antibody protection [48]. Concurrently, cellular evaluation relies on Enzyme-Linked ImmunoSpot (ELISpot) assays to screen T-cell response breadth. Intracellular Cytokine Staining (ICS) with multicolour flow cytometry further analyzes CD4^+^ and CD8^+^ T-cell polyfunctionality and memory phenotypes.

Vaccine evaluation is now transitioning toward a more comprehensive analytical framework. The integration of transcriptomic and proteomic datasets facilitates the identification of molecular signatures that predict long-term protection [49]. In addition, evaluating mucosal immunity and using single-cell B-cell Receptor (BCR)/T-cell Receptor (TCR) Repertoire sequencing have become pivotal. In the context of next-generation germline-targeting vaccines, longitudinal tracking of specific B-cell lineages provides definitive evidence of successful naive precursor priming in vivo [31].

## Clinical immunogenicity assessment and correlates of protection

### Humoral immune responses: from titers to functionality

#### Total binding antibodies and ELISA evaluation

Total binding antibodies serve as a pivotal metric for evaluating the magnitude, breadth, and durability of humoral immunity [50]. ELISA is utilized to achieve precise capture of these serological profiles, due to its high-throughput capacity and quantitative precision. (Figure 1(A)) [14]. Clinically, ELISA's primary role was evaluating antigen-specific humoral immune responses. For instance, ELISA confirmed robust gp120-binding responses in the AIDSVAX B/E trial [8,15] and recorded a dramatic geometric mean concentration (GMC) escalation in the Mosaico trial [24]. However, both trials failed to confer protection because the induced antibodies lacked neutralizing breadth and sufficient Fc-mediated effector functions [51]. Thus, modern binding antibody evaluations prioritize identifying specific functional epitopes rather than merely assessing total binding magnitude. A critical milestone in this evolution was the evaluation of V1V2-directed antibodies. Located at the HIV-1 Env trimer apex, the highly variable V1V2 loops sterically shield underlying conserved epitopes [52]. Because highly dynamic native V1V2 conformations render linear peptides ineffective for capturing functional antibodies, researchers utilized murine leukemia virus (MLV) gp70-V1V2 fusion proteins to stabilize native loop folding for precise evaluation via ELISA and other binding assays (Figure 2(A)) [53]. Utilizing these optimized antigens, the landmark RV144 trial demonstrated that V1V2-directed IgG (particularly the IgG3 subclass) served as an inverse Correlate of Risk (CoR) [14,48,50]. Systems serology elucidated that these antibodies confer protection via potent Fc-effector functions, while sieve analyses of breakthrough infections corroborated this by revealing selective pressure on V2 genetic signatures at positions 169 and 181 [54,55]. Conversely, in the HVTN 505 trial, epitope-specific ELISA elucidated the failure mechanism: while the vaccine elicited 100% gp120-specific IgG, the critical V1V2-IgG response was merely 18–20%. Furthermore, competitive blocking evaluations revealed disproportionately high Env-specific IgA, which can antagonize IgG's protective ADCC functions by competing for the same V1V2 epitope binding sites without mediating FcγR-dependent effector cell activation [9,15].

**Figure 1.**
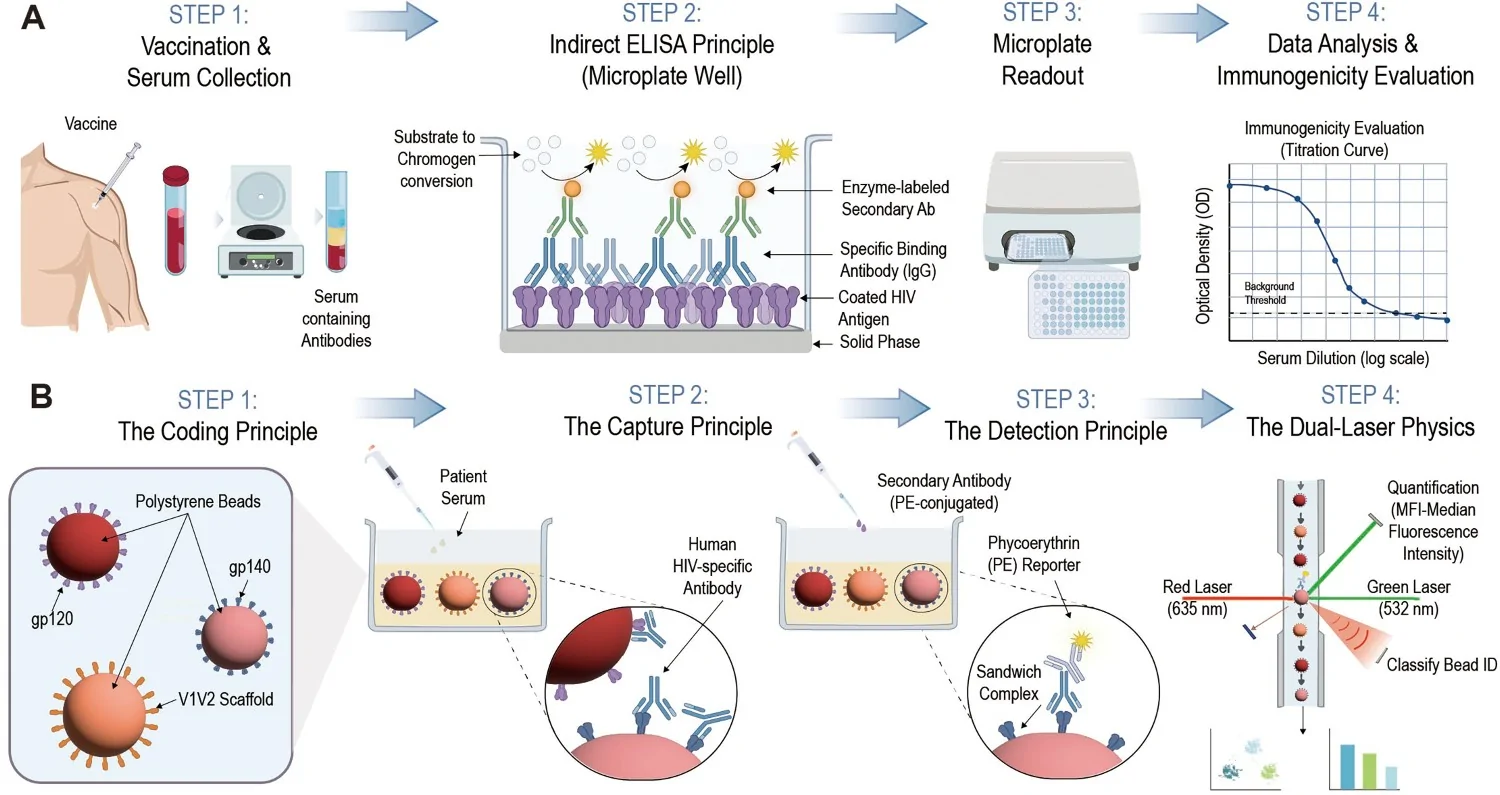
Procedural workflows and principles of indirect ELISA and multiplex immunoassay for HIV-1 vaccine immunogenicity evaluation. (A) Indirect ELISA workflow. Step 1: Blood is collected from immunized subjects and centrifuged to isolate antibody-containing serum. Step 2: Specific IgG antibodies in the serum bind to plate-coated HIV antigens. They are detected by enzyme-labeled secondary antibodies that catalyze a chromogenic substrate conversion. Step 3: The colour intensity of each well is measured as Optical Density (OD) using a microplate spectrophotometer. Step 4: OD values are plotted against log-scaled serum dilutions to generate titration curves and determine endpoint titres above the background threshold. (B) Bead-based multiplex immunoassay (BAMA/Luminex) workflow. Step 1: Distinct sets of polystyrene beads are internally colour-coded and surface-functionalized with specific HIV-1 antigens (gp120, gp140, and gp70-V1V2 scaffolds). Step 2: Antigen-specific antibodies in the serum sample are captured onto the corresponding bead surfaces. Step 3: PE-conjugated secondary antibodies bind to the captured IgG, forming fluorescent immunoassay sandwich complexes. Step 4: In a fluidic system, a Red Laser (635 nm) interrogates the bead core to classify the bead ID (antigen type), while a Green Laser (532 nm) excites the surface PE reporter to quantify antibody levels based on Median Fluorescence Intensity (MFI).

**Figure 2.**
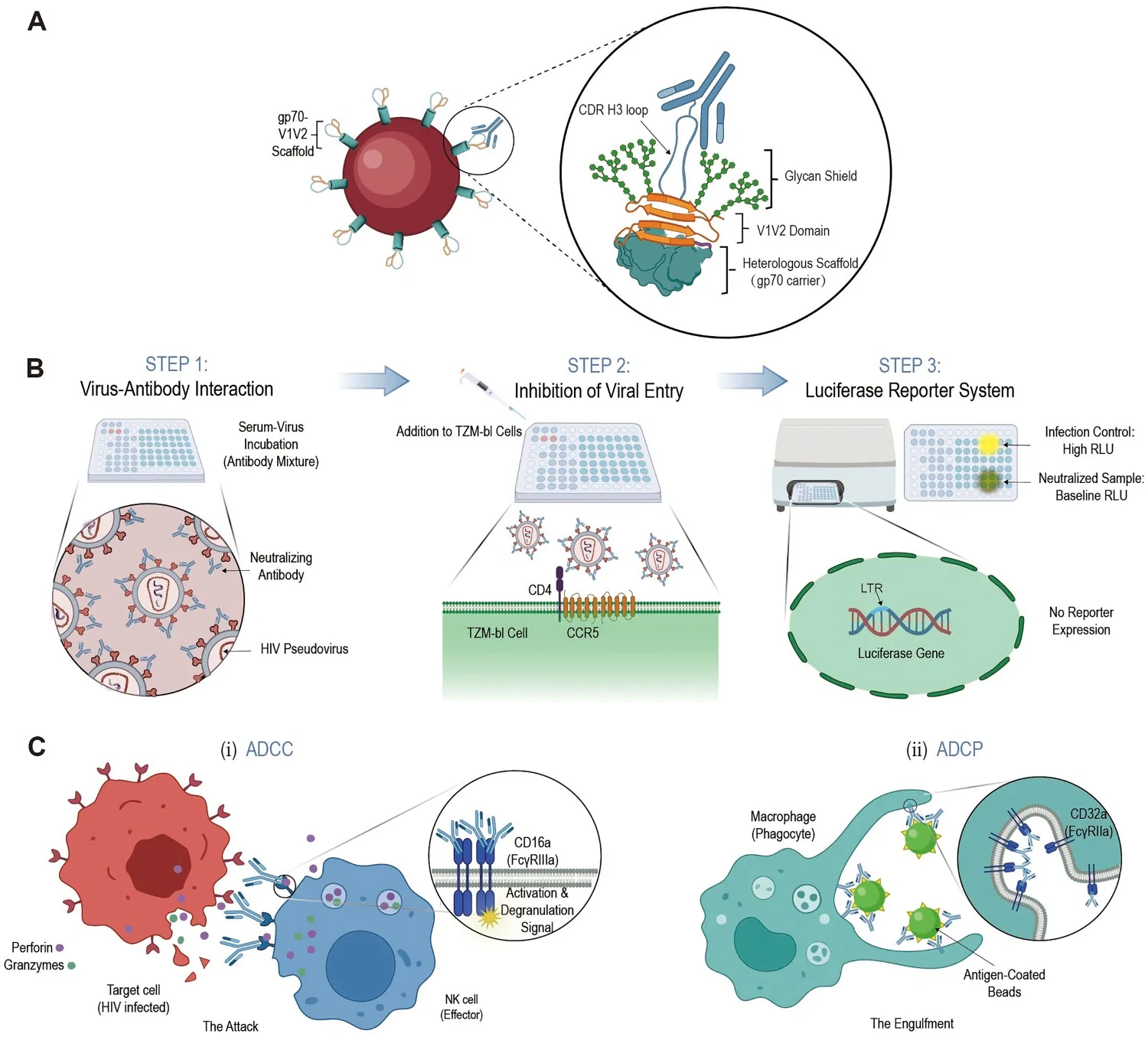
Molecular recognition mechanisms and functional evaluation assays of HIV-1 vaccine-induced antibodies. (A) Structural presentation of the gp70-V1V2 scaffold. Left: Schematic of the multivalent presentation of engineered gp70-V1V2 chimeric proteins on a carrier sphere (red). Right inset: Molecular view of antibody recognition, showing an elongated CDR H3 loop penetrating the dense glycan shield (green) to bind the stabilized V1V2 domain (orange) supported by the heterologous MuLV gp70 carrier (teal). (B) TZM-bl pseudovirus neutralization assay workflow. Step 1: HIV-1 pseudoviruses are incubated with serum containing neutralizing antibodies to allow for epitope binding. Step 2: The TZM-bl cells is added to mixture; potent neutralizing antibodies block the viral envelope from interacting with host receptors (CD4 and CCR5), thereby preventing membrane fusion and viral entry. Step 3: In the infection control, viral entry drives HIV LTR promoter-mediated luciferase gene expression, yielding high Relative Light Units (RLU). In neutralized samples, blocked entry results in no reporter expression and baseline RLU levels. (C) Evaluation of Fc-mediated antibody effector functions. (a) ADCC: Antibodies bridge an HIV-infected target cell and an NK cell. Binding of the antibody Fc domains to CD16a (FcγRIIIa) triggers an activation and degranulation signal, inducing the directional release of perforin and granzymes to lyse the target cell. (b) ADCP: Macrophages recognize antigen-coated beads via the specific interaction between membrane CD32a (FcγRIIa) receptors and the antibody Fc tails, initiating pseudopod extension and target engulfment.

#### Binding antibody multiplex assay

By utilizing diverse antigen-coupled fluorescent microspheres, BAMA generates a multidimensional Median Fluorescence Intensity (MFI) matrix reflecting antibody magnitude and breadth from minimal sample volumes (Figure 1(B)). While it demands meticulous antigen-coupling optimization and advanced bioinformatics, its high-throughput capacity and sensitivity facilitate critical standardization across large-scale cohorts. However, as BAMA measures binding rather than function, it needs to be integrated with neutralization assays to construct a robust evaluation framework [46].

Building upon the RV144 findings, Moodie et al. introduced the “V1V2 breadth score” to quantify cross-clade specificities, subclass distributions, and response rates [25]. Applying this metric, the HVTN 124 Phase I trial utilized a highly customized BAMA/Luminex platform with a 16-antigen heterologous gp70-V1V2 panel. Results demonstrated that the prime-boost strategy elicited superior IgG3 V1V2 response rates (60%), with a median breadth score over 5.9-fold higher than comparative historical regimens [26]. This methodology underscores the necessity of employing multi-subtype panels to evaluate functional antibody breadth. Similarly, in the HVTN 100 and Imbokodo trials, researchers utilized BAMA to verify that the regimens elicited broad V1V2-specific IgG3 cross-reactivity, serving as critical gating criteria to assess whether the mosaic vaccines achieved their intended broad viral coverage before advancing to efficacy trials [9]. In the IAVI G001 germline-targeting trial, researchers employed wild-type eOD-GT8 and CD4-binding site (CD4bs) knockout mutants as multiplexed probes. Subtractive analysis directly quantified specific CD4bs precursor responses, providing definitive evidence of targeted B-cell priming [31]. This precision strategy was similarly utilized in the HVTN 302 trial evaluating mRNA-encoded trimers. By incorporating “base-epitope-knockout” probes, BAMA conclusively demonstrated that membrane-anchored designs significantly diminished unwanted binding to non-neutralizing base epitopes [56].

#### Antibody binding quality and kinetics

ELISA and BAMA merely quantify antibody titres and cross-reactivity, lacking the real-time dynamics needed to fully characterize binding quality. Therefore, real-time optical sensors like surface plasmon resonance (SPR) and high-throughput multi-well biolayer interferometry (BLI) serve as essential supplementary evaluation tools [57]. By continuously monitoring antigen–antibody association and dissociation, these technologies extract kinetic parameters critical for predicting protective efficacy and validating immunization strategies. For instance, SPR and avidity analyses in the RV144 trial revealed that vaccine-induced high-affinity antibodies against the HIV V1V2 region directly correlated with reduced infection risk [55]. This kinetic profile provides biochemical evidence explaining the superiority of RV144 over standalone protein subunit vaccines like VAX003/004 [58]. Conversely, post-hoc analyses of trials like HVTN 702 used kinetic data to mechanistically attribute clinical failure to high antibody titres lacking high-quality binding affinity [19]. Furthermore, conventional titres are inadequate for evaluating modern germline-targeting vaccines aimed at broadly neutralizing antibodies. Researchers rely on SPR or BLI to accurately measure monoclonal precursor affinity and track B cell affinity maturation, confirming whether prime-boost strategies successfully drove somatic hypermutation for robust antigen recognition [31]. Mechanistically, an antibody's dissociation rate indicates its effector function potential. High-affinity antibodies with low dissociation rates maintain prolonged binding to viral or target cell surfaces to form stable immune complexes. This enduring stability is a biophysical prerequisite for inducing steric hindrance and cross-linking Fcγ receptors on natural killer cells or macrophages to trigger Fc-mediated ADCC and ADCP functions [59].

#### Neutralizing antibodies and TZM-bl assays

Eliciting bnAbs against conserved epitopes remains the ultimate goal [60], though viral defenses include glycan shielding, masking, and instability. During the process of HIV-1 Nab generation, the host's precursor B-cells must undergo complex somatic hypermutation to mature into effective bnAbs [61]. Standardized TZM-bl assays benchmark neutralizing potency by measuring luciferase reduction (Figure 2(B)) [62]. This platform offers high throughput, reproducibility, and standardization [63], but its non-physiological overexpression of CD4 and CCR5 receptors limits sensitivity to certain neutralizing antibodies compared to PBMC or A3R5 cell assays [47]. To comprehensively evaluate the true cross-reactive breadth and potency of vaccine-elicited antibodies, it is critical to utilize standardized global Env pseudovirus panels [64]. These carefully curated panels capture the vast genetic and geographic diversity of the HIV-1 epidemic, ensuring that neutralization is accurately benchmarked against clinically relevant Tier 2 strains, rather than highly sensitive but non-predictive Tier 1 laboratory strains [65].

In the HVTN 505 trial, nAb response rates were only 2.5%–27.5% and strictly confined to Tier 1 laboratory strain [9]. Similarly, HVTN 100 [64] and Imbokodo [23] confirmed that eliciting only Tier 1 nAbs cannot prevent infection, indirectly reinforcing the importance of non-neutralizing functions. Mosaic trial underscored the neutralization bottleneck: despite Ad26 immunity reducing antibody magnitude, the seronegative subgroup's efficacy was −0.7%. This demonstrates that non-protective binding antibodies, not vector delivery, were the cause of the failure, requiring new bnAb induction strategies [24]. The AMP study utilized TZM-bl assays to quantify the *in vivo* functionality of infused VRC01 antibodies [28]. The predicted serum neutralization 80% inhibitory dilution titre (PT80) biomarker demonstrated the need for multi-bnAb combinations by revealing an “all-or-nothing” protective pattern limited to extremely sensitive strains [27,28]. For active immunization, the eOD-GT8 germline-targeting strategy utilized highly sensitive pseudoviruses to detect weak precursor B-cell priming, validating the necessity for subsequent boosts with natively conformed immunogens [31]. Recently, neutralization assays revealed that a membrane-anchored trimeric vaccine elicited an 80% nAb response against a challenging Tier 2 virus, performing significantly better than the soluble trimeric form, confirming the superiority of the mRNA-LNP platform [66].

#### Fc-Mediated effector functions: ADCC and ADCP

Beyond neutralization, Fc-mediated effector functions are critical for early antiviral defense and mucosal barrier protection. In ADCC, antibody Fc regions recruit natural killer (NK) cells to directly lyse HIV-1-infected targets (Figure 2(Ci)). ADCP utilizes myeloid cells (macrophages, monocytes, and dendritic cells) to engulf targets and subsequently present antigens to prime T-cell responses (Figure 2(Cii)) [67]. ADCC assays have evolved from traditional ^51^Cr release assays [68] to high-sensitivity flow cytometry and real-time luciferase reporters [69]. This technical evolution is complemented by the transition to target cells expressing native-like trimers, primary CD4^+^ T cells rather than gp120-coated models, with CD4-mimetics utilized to fully expose concealed basal epitopes [70]. For ADCP, flow cytometry directly quantifies engulfment [71], while high-throughput NFAT reporter systems now enable simultaneous ADCC and ADCP screening [72]. Imbokodo study evaluated ADCC and ADCP as protective pathways independent of bnAbs. Given ADCP’s role as a key CoR in non-human primate(NHP) Simian-Human Immunodeficiency Virus (SHIV) models, human validation was essential [73]. Imbokodo successfully induced robust ADCP and protective-trending, ADCC-mediating IgG3 V1V2 antibodies; however, the lack of efficacy demonstrated these functions were not independent CoRs [23]. This highlights that robust Fc-mediated effector arms alone cannot protect high-risk populations, necessitating optimization for broader multi-arm immune synergy.

Notably, preclinical models suggest that ADCP can paradoxically induce localized immunosuppression within complex microenvironments. FcγR-mediated phagocytosis can activate the AIM2 inflammasome in macrophages, thereby upregulating Programmed Death-Ligand 1 (PD-L1) and Indoleamine 2,3-dioxygenase (IDO). This can result in the dampening of NK and T-cell functions. Although there is currently a lack of direct evidence from human trials, this evidence suggests HIV vaccine clinical immunogenicity assessments should not only quantify the magnitude of ADCP, but also monitor shifts in macrophage phenotype to ensure that vaccine-induced Fc-effector functions do not inadvertently compromise overall cellular immunity [74].

### Cellular immune responses: phenotyping and polyfunctionality

#### ELISpot

The ELISpot assay serves as the standard high-throughput tool for screening HIV-1 T-cell responses, quantifying repertoire magnitude via single-cell interferon-gamma (IFN-γ) Spot Forming Cell (SFC) enumeration (Figure 3(A)) [75]. Its sensitivity detects rare T-cells without expansion, preserving native effector profiles. Consequently, its efficiency and scalability make it a premier alternative to flow cytometry for initial cellular evaluation [76]. However, the conventional IFN-γ ELISpot assay is constrained by a quantitative bottleneck at high response frequencies due to spot merging, alongside an inability to phenotypically identify specific responding cell subsets. Furthermore, relying on a single cytokine marker often fails to accurately predict in vivo protective immunity, which typically requires polyfunctional T-cell responses. Finally, the assay's high technical variability and complex baseline noise present significant standardization and statistical challenges when distinguishing true vaccine-induced responses [75,76].

**Figure 3.**
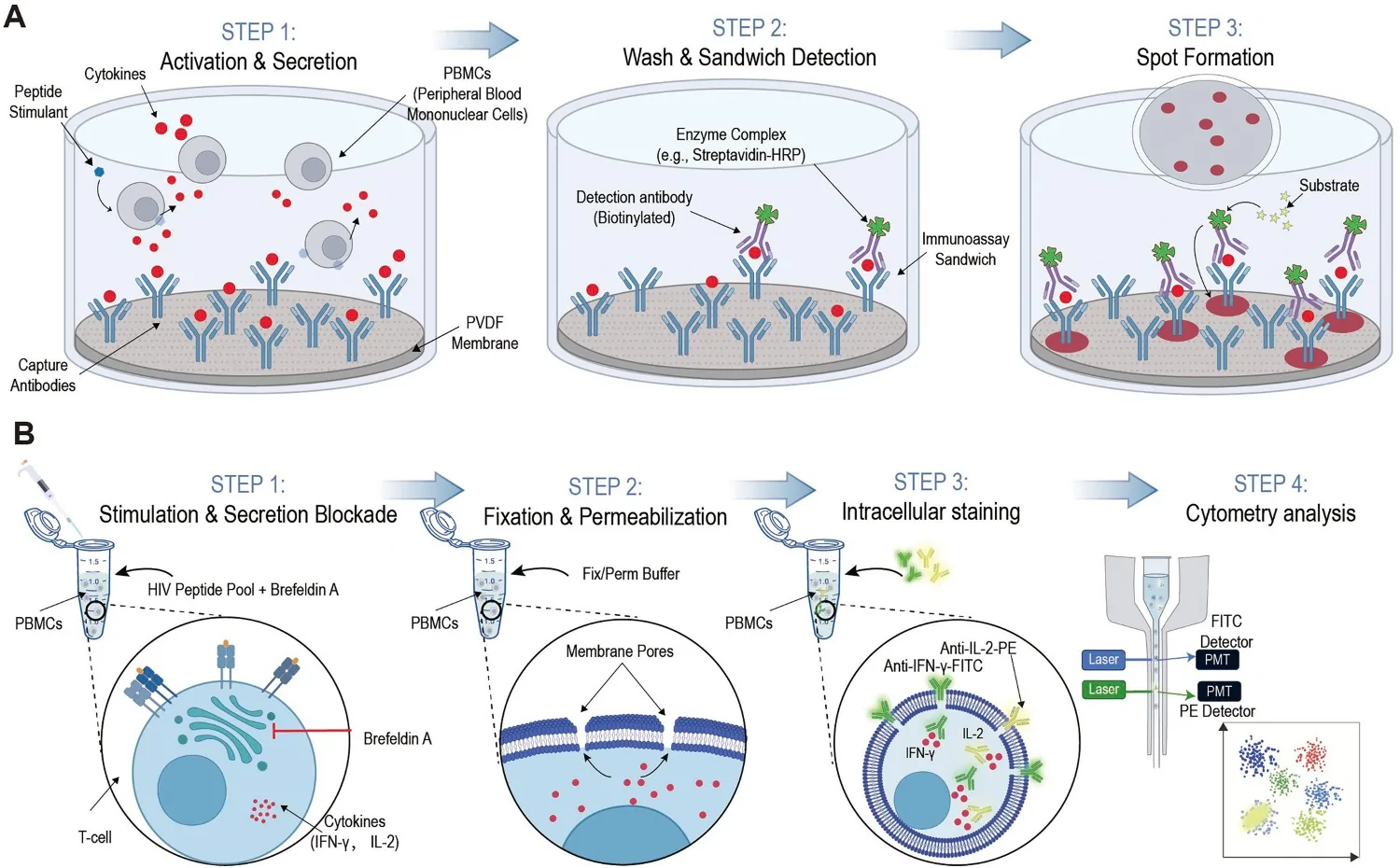
Methodological workflows of ELISPOT and ICS assays for T-cell immunogenicity profiling. (A) ELISPOT assay workflow. Step 1: PBMCs are cultured on an antibody-coated PVDF membrane and stimulated with a specific peptide pool. Antigen-specific T-cells respond by secreting cytokines (e.g. IFN-γ), which are immediately captured in situ by the underlying antibodies. Step 2: Following a wash step to remove cells, biotinylated detection antibodies and streptavidin-HRP enzyme complexes are added to form a stable immunoassay sandwich. Step 3: A chromogenic substrate is introduced and enzymatically converted into insoluble precipitates, generating distinct, visible spots on the membrane where each spot represents a single cytokine-secreting cell. (B) ICS and flow cytometry workflow. Step 1: PBMCs are stimulated with an HIV peptide pool in the presence of Brefeldin A. Brefeldin A inhibits the Golgi-mediated secretory pathway, forcing newly synthesized cytokines (such as IFN-γ and IL-2) to accumulate inside the T-cells. Step 2: Cells are treated with a Fix/Perm buffer to cross-link cellular proteins and generate microscopic membrane pores, facilitating the entry of macromolecular reagents. Step 3: Fluorophore-conjugated antibodies (Anti-IFN-γ-FITC and Anti-IL-2-PE) pass through the permeabilized membrane to specifically target and bind the trapped intracellular cytokines. Step 4: The stained single-cell suspension is fluidically routed past dual lasers. Photomultiplier tube (PMT) detectors register the specific fluorescence signals, allowing for multiplexed phenotypic and functional quantification on a cytometry dot plot.

Clinically, ELISpot immunogenicity correlates non-linearly with protective efficacy. In the STEP trial, robust ELISpot magnitudes paradoxically coincided with absent protection and elevated infection risk [12]. This establishes that IFN-γ intensity signifies immune activation rather than protection, driving a shift toward ICS to assess T-cell polyfunctionality. Similarly, using a dual-peptide strategy, the HVTN 503 trial revealed that 77.4% of Clade B vaccinees robustly recognized heterologous Clade C antigens [11]; however, this theoretical cross-clade breadth failed to yield clinical efficacy. In RV144 trial, a modest 19.7% T-cell response contrasted sharply with the landmark 31.2% protective efficacy. By excluding cellular immunity as the primary driver, this discrepancy redirected focus toward protective humoral mechanisms [14].

#### Intracellular cytokine staining

Via peptide stimulation, Golgi blockade, and marker labelling, ICS distinguishes CD8^+^ cytotoxic from CD4^+^ helper responses and identifies polyfunctional T-cells hypothesized for HIV-1 containment (Figure 3(B)) [77]. Utilizing ICS, the STEP trial revealed high levels of CCR5^+^ CD4^+^ target cells in subjects with pre-existing rAd5 immunity [78], explaining the paradox of robust ELISpot reactivity alongside an increased risk of infection in a subset of participants. In RV144, ICS redefined the regimen as a CD4^+^ helper vaccine (34.0% Env-specific CD4^+^ vs. 7.6%–11.1% CD8^+^), facilitating non-neutralizing antibodies. Conversely, HVTN 505 failed despite high T-cell frequencies, proving magnitude without precise epitope targeting is futile [9]. HVTN 100 integrated ICS with the computational framework for unbiased combinatorial polyfunctionality analysis of antigenspecific T-cell subsets (COMPASS) framework, confirming Env-specific CD4^+^ enhancements to advance to HVTN 702 [18,79]. In Ad26 mosaic trials, ICS confirmed broad reactivity, though clinical failure indicated such breadth remains insufficient against global diversity.

Currently, ICS enables high-resolution screening for next-generation vaccines. IAVI G001 utilized dual-fluorescence differential staining (eOD-GT8 vs. CD4-binding site knockout mutant eOD-GT8-KO11) and single-cell BCR sequencing to confirm VRC01-class precursor priming [31]. For mRNA platforms, ICS identified a tailored circulating follicular helper T-cells (cTfh, expressing IL-21/Th2) with quiescent CD8^+^ responses, optimizing affinity maturation while preventing premature APC clearance [80]. Finally, HVTN 124 utilized Potential T-cell Epitope (PTE) pools to substantiate a 100% CD4^+^ seroconversion rate and high-quality polyfunctional subsets, providing a paradigm for assessing immunity against viral evolution [26].

#### Lymphocyte proliferation assay

Unlike ELISpot and ICS, which capture phenotypes and cytokine profiles but cannot validate sustained memory kinetics, the Lymphocyte Proliferation Assay (LPA) assesses mitotic memory expansion. Following *ex vivo* HIV-1 antigen re-challenge of isolated PBMCs, clonal expansion is quantified via ^3^H-thymidine incorporation or CFSE dilution to derive the Lymphocyte Stimulation Index (LSI) [81]. Robust LSI reflects strong antigenic recognition and a substantial proliferative reservoir. In RV144, despite lacking vigorous CD8^+^ activity, LSI escalated from baseline to peak values of 24–32, underscoring high-quality CD4^+^ helper responses [48,81] and mechanistically explaining facilitated antibody affinity maturation and emergent protective efficacy.

## Novel exploratory assays

### Mucosal local immune evaluation

Since over 90% of HIV-1 infections occur mucosally, standard peripheral blood assessments fail to recapitulate local defenses [82]. Studies show a strong positive correlation between Env-binding IgG, including V1V2-specific titres, in serum and mucosal secretions. This reveals that mucosal IgG is not actively produced in situ, but is primarily a passive transudate leaking from the systemic circulation [83]. Consequently, high peripheral blood IgG titres merely predict the magnitude of systemic transudation and fail to reflect whether a dedicated, self-sustaining immune defense has been established at the mucosal portal of entry [83]. Due to the prohibitive clinical feasibility of comprehensive mucosal sampling, the process of conducting a genital or intestinal biopsy is technically demanding, is subject to high inter-individual variability, and yields extremely low cell numbers that frequently preclude downstream functional assays; contemporary mucosal assessment methodologies frequently prove incapable of accurately predicting local protective efficacy. The processing and culturing of solid mucosal tissues, such as cervical explants, has been demonstrated to disrupt natural in vivo tissue trafficking signals, resulting in the progressive down-regulation of key tissue retention markers like CD69 over time. The field of vaccine research continues to place significant reliance on peripheral blood evaluations [84].

Humoral evaluations of secretions eluted from Weck-Cel sponges have shifted to specific activity (SA) models to overcome dilution errors, utilizing BAMA to normalize antigen-specific antibody signals against total local immunoglobulin concentrations [83]. To precisely separate and quantify sparse mucosal IgA without IgG cross-isotype interference, samples must first be depleted of IgG using Protein G plates prior to specific IgA measurement [83]. Additionally, tracing the compartmental penetration and functional activity of systemically administered broadly neutralizing antibodies, such as VRC01, into genital and rectal secretions remains a critical strategy for evaluating mucosal protection [85]. Cellularly, evaluating local protection relies on isolating sentinel T_RM_ by enzymatically digesting solid biopsies with collagenase IV and mechanical dissociation to yield viable single-cell suspensions [84]. Biopsy flow cytometry accurately distinguishes T_RM_ cells using core retention markers while simultaneously tracking HIV-1 susceptibility co-receptors and integrins to ensure vaccines do not inadvertently increase viral targets at the epithelial barrier [83,84]. Furthermore, spatial dynamics can be mapped without tissue dissociation using RNA in situ hybridization (RNAscope) combined with CD69 immunohistochemistry, which offers anatomical correlates by accurately localizing vRNA + infected cells within distinct layers such as the epithelium and submucosa [84].

### High-dimensional immune profiling

Traditional vaccine assessments predominantly use neutralizing antibody titres as primary surrogate endpoints; systems serology integrates functional assays with high-throughput biophysical profiling (e.g. subclasses, Fc affinities, glycosylation) to deconstruct polyclonal responses [86]. Using machine learning (ML) algorithms like multivariate regression, partial least squares discriminant analysis (PLSDA), and least absolute feature shrinkage and selection operator (LASSO), researchers can distil predictive immune fingerprints from high-dimensional matrices [86,87]. For instance, this approach successfully distinguished the highly functional, V1V2-specific IgG3 protective mechanisms in the RV144 trial from the poorly functional IgG4-driven responses in the non-efficacious VAX003 trial [87]. Advanced computational immunology is integral to subsequent studies like HVTN 505 and the AMP trials [88]. ML models actively decode complex B-cell repertoires and map the SHM trajectories required for germline-targeting regimens [89]. Furthermore, multi-task learning (MTL) and deep learning computationally predict effector functions and neutralization breadth directly from clinical viral envelope sequences [88].

However, the integration of machine learning (ML) and single-cell multi-omics is impeded by a significant challenge, namely the “curse of dimensionality” that arises from the analysis of high-dimensional data in small-scale clinical trials. The processing of a large number of biophysical features against a limited number of participants can result in a phenomenon known as “dimensionality explosion”. In the absence of rigorous feature selection and regularization, models are susceptible to severe overfitting, resulting in the capture of cohort-specific noise rather than authentic biological correlates [89]. Consequently, such models frequently demonstrate inadequate generalizability; for instance, HIV-1 resistance prediction models are afflicted by severe subtype biases, exhibiting optimal performance on prevalent Clade B/C sequences but substandard performance on underrepresented clades [88]. Finally, routine implementation of single-cell multi-omics in large trials remains severely constrained by sample throughput bottlenecks and high costs [86].

### B cell repertoire profiling and monoclonal antibody validation

Contemporary B-cell profiling utilizes differential flow cytometry strategies, specifically wild-type alongside base-knockout probes, to establish a rigorous gating logic that isolates effective neutralizing clones from off-target basal responses [31]. Furthermore, evaluating germinal center (GC) reactions provides a critical molecular readout of ongoing affinity maturation [90]. Post-sorting single-cell BCR sequencing (scBCR-seq) captures paired heavy-light chain data to screen germline precursors and reconstruct SHM trajectories [90,91]. The clonal evolution of these lineages is routinely mapped using phylogenetic clustering tools, such as the treeC1 package, before in vitro monoclonal antibody functional assays solidify the genotype-phenotype-function causal chain [28].

Crucially, evaluating next-generation germline-targeting vaccines fundamentally differs from conventional single-immunogen approaches [90]. Because the core of germline-targeting relies on multi-dose sequential immunization, the evaluation focus shifts from endpoint antibody titres to precisely tracking intermediate B-cell lineage evolution nodes. Underscoring this paradigm, Steichen et al. and Marchitto et al. recently achieved bnAb elicitation in nonhuman primates via germline-targeting strategies. Ultimately, fusing structural probes with high-throughput single-cell omics provides the definitive molecular-level guidance needed to track these intermediate nodes and validate sequential regimens [90,91].

## Regulatory science and inter-laboratory standardization

To translate immunogenicity data into comparable biomarkers, evaluation methods must strictly comply with the ICH M10 “Bioanalytical Method Validation” guideline and directives from regional regulatory authorities [92]. Analytical standardization requires laboratories to benchmark assays using well-characterized reference reagents and globally representative standardized Tier 2 pseudovirus reference panels to eliminate systemic biases across platforms from different centres [63]. For complex vaccination strategies, clinical trials involving multiple centres must achieve harmonization across laboratories through formal PT (Proficiency Testing) programmes. In the standardized TZM bl pseudovirus neutralization assay, participating laboratories globally must achieve a high degree of concordance within a predefined, strict acceptable threefold variation range [47]. Similarly, for cellular immune assays like ICS and ELISpot, the MIATA (Minimal Information About T Cell Assays) guidelines mandate the disclosure of key details, such as sample processing and background subtraction algorithms, fundamentally eliminating “technical black boxes” between laboratories [93].

While traditional evaluation platforms have established mature validation systems, cutting edge technologies with high dimensionality like high throughput sequencing and single cell multiomics still face unique validation challenges when entering the regulatory submission pathway. The core difficulty lies in proving to regulatory agencies that “dry lab” data processing is not a “black box”; every analytical step in bioinformatics must be fully traceable and satisfy Data Integrity ALCOA (attributable, legible, contemporaneously recorded, original or a true copy, and accurate) principles [94], exactly like wet lab SOPs and laboratory notebooks. Unrecorded data loss during bulk processing, such as bypassing Biopython data type errors, directly causes downstream antigen epitope predictions to misalign. Furthermore, regulators require the implementation of widely validated and version locked standardized tools to prevent erroneous conclusions resulting from discrepancies in underlying code logic [95].

## Challenges in defining correlates of protection

Unfortunately, after four decades of exploration, the scientific community has yet to identify a universally applicable correlate of protection (CoP) for HIV vaccines, a failure that profoundly reflects the extreme genetic diversity of the virus and the intricate complexity of host immune responses [96]. When evaluating clinical efficacy, we must strictly distinguish between correlates of risk (CoR) observed in specific cohorts and true CoPs capable of predicting defensive mechanisms [97]. While traditional vaccines rely on high-titre functional neutralizing antibodies as unambiguous mechanistic CoPs (mCoPs), interpreting HIV vaccine data frequently falls into the logical pitfall of mistaking a CoR for an mCoP [96–98]. For example, binding antibodies targeting the V1V2 region and high-titre gp120 antibodies demonstrated a statistical inverse correlation with infection risk in the RV144 and VAX004 trials, respectively [8,48,96]. However, restricted by extremely weak neutralizing activity and marginal overall trial protection, these markers represent unvalidated inverse CoRs that may merely reflect unalterable innate host immune characteristics or genetic resistance rather than true protection induced by vaccination [96,98]. Furthermore, systemically measured candidate CoPs must effectively represent actual mucosal defensive capabilities given the predominant mucosal transmission routes of HIV. The validation of potential markers remains an arduous endeavour compounded by antigenic disparities between sensitive laboratory strains and circulating viruses alongside the dynamic waning of protective efficacy over time [96]. Until definitive in vitro mechanistic evidence and independent cohort validations are achieved, the scientific community must objectively define CoR limitations and exercise extreme caution regarding statistical associations to ensure the rigorous advancement of HIV vaccine development.

## Conclusion

Precise immunogenicity frameworks are foundational for deciphering HIV-1 evasion and ending the pandemic. Driven by viral diversity and elusive protection, clinical assessment has shifted from basic serology to high-dimensional profiling. Post-RV144, AMP, and HVTN trials, evaluation transcends neutralizing antibodies by integrating BAMA, Fc-effector profiling, and polyfunctional T-cell ICS. Characterizing non-neutralizing functions and V1V2 responses has clarified partial protection, informing candidate prioritization. However, lacking universal CoPs hampers efficacy projections. Furthermore, peripheral blood reliance creates an anatomical disconnect from localized defenses, such as lymph node GC affinity maturation or mucosal T_RM_. Future research should integrate single-cell multi-omics and systems biology to distil predictive “immune fingerprints.” Amid mRNA and germline-targeting advances, evaluation must pivot toward longitudinal BCR ontogeny and in situ mucosal immunity. Ultimately, this framework remains the scientific basis for overcoming HIV-1 evasion and achieving the global AIDS-free mission.
